# The problem with disclosing IVF status: Normative parental timelines and the gendered support needs of involuntary childless heterosexual couples undergoing in vitro fertilisation

**DOI:** 10.1177/13591053251344713

**Published:** 2025-06-19

**Authors:** Eva Prikrylova, Sarah Seymour-Smith, David Wilde, Maria Kontogianni, James Hopkisson, Nicholas Raine-Fenning

**Affiliations:** 1 Nottingham Trent University, UK; 2 University of Nottingham, UK

**Keywords:** infertility, disclosure of infertility, gender roles, IVF/ICSI, social support

## Abstract

Subfertility and infertility are stigmatising conditions impacting on the gender identity of its sufferers. Consequently, heterosexual couples undergoing fertility treatment find it difficult to disclose their condition. While research suggests that fertility treatment is an isolating and stressful experience for both partners, there is sparse literature investigating what support mechanisms exist. This study interviewed 10 heterosexual couples undergoing IVF or IVF/ICSI to explore how they constructed infertility and negotiated disclosure and support. Data were analysed using a social constructionist informed Thematic Analysis. Three themes were identified: ‘*The next step’: Omnipresence of normative parental timelines; Balancing the need for social support with problems associated with disclosure;* and *Men’s supportive role*. We argue that barriers to disclosure hinder support needs and that strategies to negotiate this would be useful to infertility patients.

## Introduction

Infertility affects one in seven heterosexual couples in the UK – approximately 3.5 million people and is defined as the inability to conceive within 12 months of having regular unprotected sex (National Health Service, 2020). The cause of infertility is equally attributed across gender, 30% is caused by a male factor, and 30% by a female factor, the remaining 40% of cases being unexplained or attributable to both parties (The American Society for Reproductive Medicine, 2017). Gnoth et al. (2005) highlight the confusion of medical terminology – whilst infertility may be associated with sterility due to the low chance of spontaneous pregnancies, the term subfertility encapsulates any form of reduced fertility resulting in delayed or unwanted lack of conception. Both infertility and subfertility can be treated by infertility treatment.

### Social construction of infertility across cultures

Infertility is characterised by the absence and desire for a child. The way individuals make sense of infertility varies cross-culturally due to the socially constructed nature of parenthood and what infertility means for that culture (Greil et al., 2010). Greil et al. (2010) argued that in the Global North women’s voluntary childlessness is socially acceptable, as they can pursue other goals such as education and careers. Within stronger patriarchal systems (e.g. sub-Saharan Africa, South Asia, Middle East), women’s main fulfilment revolves around motherhood (Inhorn, 2003a). Childbearing in these societies is seen as mandatory (Inhorn, 2003b; Van Balen, 2008). In some countries the rules around marriage mean that men can divorce a woman if she does not bear a child. A childless woman occupies the lowest societal level, often ostracised and isolated by their community, abused by her husband and his family (Inhorn and Patrizio, 2015; Van Balen, 2008; Van Balen and Bos, 2009). Divorced childless women are frequently left living in poverty (Van Balen and Bos, 2009).

In some cultures (e.g. some poor areas of the Indian subcontinent and Sub-Saharan Africa), male infertility is still not recognised meaning that women take the blame (Inhorn, 2003a; Van Balen and Bos, 2009). When male infertility is acknowledged, the condition is viewed as highly stigmatising. Thus, infertile men often refuse infertility tests and allocate the blame to the woman (Inhorn, 2003b). So, while infertility in the Global North impacts mostly individuals, sufferers in low resource countries are impacted at individual, community, social, religious and economic levels (Van Balen, 2008; Van Balen and Bos, 2009).

The above review highlights both the socially constructed nature of infertility and the impact of this upon marital relationships, raising the importance of studying heterosexual couples (Greil et al., 2010). To situate our UK study, we next review research about routes to parenthood in the Global North.

### Route to parenthood

Research investigating reasons and decisions for becoming a mother highlight a socially acceptable order in achieving motherhood. Steps to motherhood involve securing an education, travel, career and property ownership (Budds et al., 2016; Perrier, 2013; Sevón, 2005). Social expectations regarding financial security, career progression, a stable romantic relationship and emotional maturity all need to be balanced against women’s optimal biological time for pregnancy (Budds et al., 2016; Cooke et al., 2012; Locke and Budds, 2013) resulting in a very limited window for women to become mothers.

In terms of heterosexual fatherhood, female partners play a critical role in male decision-making processes (Deslauriers, 2011; Marsiglio et al., 2000; Von Der Lippe and Fuhrer, 2004). The socially expected order for fatherhood are career, marriage, children (Hadley and Hanley, 2011). As male fertility was not seen as impacted by age, it did not result in feelings of responsibility and guilt for leaving it too late (Hadley and Hanley, 2011; Sylvest et al., 2014).

People are socialised into their reproductive story and their vision of themselves as parents long before their pregnancy and parenthood (Jaffe, 2017; Jaffe and Diamond, 2011). Infertility acts as a biographical disturbance to this life story posing the question of how heterosexual couples in fertility treatment (re)construct their reproductive story regarding parenthood.

### Gender identities and infertility

The desire to have children is socially constructed as a female biological instinct and measurement of femininity; equating womanhood with motherhood (Gillespie, 2000; Ulrich and Weatherall, 2000). Similarly, fatherhood is viewed as confirmation of masculinity; evidencing fertility and virility (Berg et al., 1991). Infertility challenges traditional masculinity (Thomas, 2018; Throsby, 2004), triggering feelings of shame and emasculation (Gannon et al., 2004; Webb and Daniluk, 1999). Thus, infertility and involuntary childlessness can lead to social stigma for both women and men (Fernandes et al., 2006) and secrecy (Kirkman, 2003.; Gannon et al., 2004; Hanna and Gough, 2020; Malik and Coulson, 2008).

### Social support within treatment

Social support is key to the wellbeing of couples undergoing fertility treatment, decreasing the likelihood of terminating the treatment (Vassard et al., 2012). A lack of perceived social support from close family was associated with treatment stress (Martins et al., 2014), anxiety, depression and health complaints (Lechner et al., 2007). Disclosure of infertility could cause unsupportive responses such as embarrassment, awkward reactions, inappropriate jokes and a general lack of empathy (Ussher and Perz, 2018). The expression of pity, problem solving advice and general insensitivity hindered a supportive relationship and led to a lack of disclosure (Abraham, 2019). In this paper the term non-disclosure denotes times when couples chose not to share information with family and friends about their difficulties conceiving and infertility treatment.

### Gender roles within treatment

Regardless of causal factors, most medical infertility procedures target women’s bodies, with medics viewing women as their primary patient (Hanna and Gough, 2020; Throsby, 2004) Thus, the male partner often feels marginalised and takes on a supportive role (Culley et al., 2013; Hanna and Gough, 2020; Malik and Coulson, 2008). However, women reported their spousal support as the most crucial during fertility treatment (Abraham, 2019; Jafarzadeh-Kenarsari et al., 2015). Spousal communication difficulties regarding treatment could lead to the termination of the treatment (Abraham, 2019; Vassard et al., 2012).

Throsby and Gill (2004) outlined four roles of the male partner within infertility treatment: providing a sperm sample; support administrating injections; veto rights over the decision to terminate treatment; and providing emotional support. However, men experienced difficulties discussing treatment, supressed their own treatment related stress and emotions, and were consequently unable to support their partner, which ultimately made their partner feel isolated and lonely (Malik and Coulson, 2008; Throsby, 2004; Throsby and Gill, 2004). Throsby (2004) and Throsby and Gill (2004) linked men’s reluctance to talk to traditional masculinities where men do things rather than talk about them. Research on male infertility online support forums (Hanna and Gough, 2016, 2017; Malik and Coulson, 2008) partially confirmed these findings. Men on the forums viewed emotional support of their woman as their main role, however some of them felt hopeless when dealing with her emotions regarding infertility (Hanna and Gough, 2017), or if she pushed them away (Malik and Coulson, 2008). However, Sauvé et al. (2020) found that some couples developed an increased closeness from undergoing infertility treatment.

### The current study

This study employs a social constructionist epistemology to explore how heterosexual couples in the UK manage disclosing their infertility status and how this links to support seeking during fertility treatment. Stigma associated with infertility is gendered but not enough is known about how heterosexual couples negotiate disclosure to receive the support that they need. Additionally, the supportive role is problematic for men and needs further investigation.

## Method

### Design

Dyadic semi-structured interviews were conducted with heterosexual couples after they finished their first treatment cycle of in vitro fertilisation (IVF; where eggs are retrieved and fertilised by sperm in a lab), or in vitro fertilisations with intracytoplasmic sperm injections (IVF/ICSI where in addition to IVF, sperm is injected directly into the egg). Since IVF is often seen as the last option within the line of existing fertility treatments, the threat of involuntary childlessness and consequent change to reproductive plans of these couples was very real. Reflexive Thematic Analysis with a social constructionist epistemology was employed to investigate co-produced accounts of fertility treatment.

### Sampling and data collection

Opportunity sampling was employed. Participants were recruited in waiting rooms at a UK/Midlands, England assisted conception unit and via their Facebook page. Only heterosexual couples who were childless before commencement of their treatment, with any type of infertility, and who were using their own eggs and sperm were included. Ten heterosexual couples (*n* = 20) were recruited. Seven couples were NHS funded and three couples were self-funded. This was the first treatment cycle of IVF or IVF/ICSI for all but two couples. Most couples had previously experienced some form of less invasive fertility treatments. The longest duration for trying to conceive was 8 years.

### Timeline of treatment and interviews

Eight couples were recruited when they attended their first clinic appointment. Couples proceeded with their treatment cycle up to embryo transfer, followed by 2 weeks wait, after which they took a pregnancy test. Interviews took place soon after they knew the results of their pregnancy test. Five couples were pregnant, three couples were not.

To achieve a larger sample, two couples were recruited via Facebook and interviewed retrospectively but within 2 years of their initial treatment, which had been successful. The study was part of a multi-study project. The duration of one treatment cycle varied between 6 and 8 weeks (from first hormonal injection to pregnancy test). The data collection took approximately 6 months.

Participants were aged 25–45 years., Eight couples were interviewed face-to-face, two couples were interviewed via Skype. Interviews ranged from 43 minutes to 2 hours and 35 minutes duration, totalling 821 minutes (approximately 14 hours).

The study involved two versions of an interview schedule depending on successful/unsuccessful treatment outcome. The schedule was informed by existing research and input from a patient participation group. The schedule was divided into eight sections: trying for a baby; pre-treatment and diagnosis; first consultation; treatment and its impact on everyday life; 2-week wait; results; review consultation; impact of the treatment. Interviews were audio-recorded.

The study was ethically approved by both the National Health Service Research Ethics Committee (NHS REC) and the host institution ethics committee. Informed consent was obtained prior to interview and couples were informed about their right to withdraw. Participants were monitored during the interview for any signs of distress and offered breaks if needed.

### Analytical position and procedure

Reflexive Thematic Analysis (TA; Braun and Clarke, 2006, 2022) was used to identify patterns of meaning within the data. The first author was an infertility sufferer and therefore took an insider stance. However, the second author was voluntarily childless and also coded the data, thus was able to member check to insure validity. We applied a social constructionist epistemology, which fits well with the notion that infertility is socially constructed. Social constructionism views knowledge as culturally and historically specific and created within social interaction (Burr, 2015) and permits examination of accepted assumptions about phenomena.

Interview recordings were transcribed and anonymised. The analysis followed the six-steps outlined by Braun and Clarke (2006, 2022). The first two authors immersed themselves in the data, listening to the recordings and re-read the transcripts. Next, the data was coded at the semantic then latent level. These codes were collated to generate themes. The meaning behind each theme was refined to address the research aim. Extracts demonstrating the core concept of each theme were identified to write the analytical section. To apply the social constructionist lens with its emphasis on discourse, we also paid attention to social actions (e.g. justifying decisions) and considered how people were positioned in the data. Finally, we investigated deviant cases – these are exceptions and irregularities within the identified thematic patterns and are useful in checking the validity of interpretations when that pattern is absent (Wiggins, 2017).

## Analysis and discussion

The analysis identified three main themes:

- ‘The next step’: Omnipresence of normative parental timelines
- Balancing the need for social support with problems associated with disclosure
- Men’s supportive role

### ‘The next step’: Omnipresence of normative parental timelines

For our participants, infertility disrupted the ‘normal/socially accepted’ life trajectory. Seven out of 10 couples (14 out of 20 participants) referenced this, usually when asked about how they decided to have a baby. A chronological order was oriented to across couples’ responses which referenced key stages such as marriage, job/financial security, home ownership and trying for a baby (Budds et al., 2016; Perrier, 2013; Sevón, 2005). The order was not strict, but elements of these stages were omnipresent across the data.

#### Extract 1: Joana/ Steve

I: I would like you to think back (laughs) these nine years, when you decided to have a baby, how did you decide to have a baby?

Joana: We’d got married hadn’t we, we’d got married in 2008.

Steve: the next step according to her

Joana: We’d got our own house and it was just natural, the next the next step. We come from big families, there’s lots of children in both of our families, isn’t there?

Steve: Breed like rabbits.

I: (laughing)

Joana: yeah our families do. We don’t ehm and

Steve: It was just the next step yeah yeah

Joana: It was just the natural next step.

I: Was it both of you the same, the meaning of having a baby?

Steve: Yes. It was natural. It was the natural thing to do, it was the right thing to do

Joana: yeah we’ve been together years, hadn’t we and

Steve: yeah we was financially stable, secure. You had your job that was stable and secure. So it was just the next natural step.

Joana and Steve discussed steps in a way that conveyed the importance of a stable base upon which to build a family; for them, a strong relationship is constructed via ‘we’ve been together for years’ and financial stability and job security laid the groundwork for the ‘next natural step’. By following this natural order, they presented themselves as responsible parents whose priority was the financial security of their unborn child and additionally justified the decision to have a child at that time in their lives as ‘it was the right thing to do’. Having a baby was co-constructed as the goal of this relationship phase. While this finding echoes other research regarding the socially expected order in motherhood and parenthood (e.g. Budds et al., 2016; Hadley and Hanley, 2011) and the concept of reproductive story and parenthood being a developmental stage marking mature adulthood (Jaffe, 2017; Jaffe and Diamond, 2011), couples in our study constructed this stage as a phase in a heterosexual romantic relationship, rather than within their personal life or adulthood.

This order is co-constructed as ‘just the natural step’ (Joana) and ‘just the natural thing to do’ (Steve). The prevalence of ‘just’, which was evident across the data, qualifies this as a minimal step. However, for infertile heterosexual couples, this next step was not simple. Joana highlights how they both come from big families, with Steve joking that they ‘breed like rabbits’, implying how easy it is for everybody around them to achieve parenthood. Jo concurs, yet also adds, ‘we don’t’. Thus, we begin to see how infertility marks out a different life trajectory and categorises the couple as different to others, which can be stigmatising (Goffman, 2009). This notion of ‘being the other’ was common in our data. Notably, the absence of a baby is a publicly visible disruption to the expected normative order, regardless of the couple’s attempts to fulfil this norm.

We had one deviant case amongst our participants. Petra and Neil referred to the natural order whilst presenting a different narrative; specifically, they postponed trying for a child.

#### Extract 2: Petra/Neil

I: So, now I would like you to think back at complete beginning when you decided to have a baby. How did this happen? Why, who, what was the reason?

Petra: Well, it’s very simple. We got married [but we said,

Neil: [we both said

Petra: Not yet, not yet, not yet [and then it got to the point

Neil: [we wanted to enjoy ourselves and then we thought ughh

Petra: where we said, [ ‘Let’s do it now’.

Neil: [‘We gonna get on with it now’,

I: (laughing)

Neil: so that was that was really it.

Petra: I wasn’t one of the people who would go, marriage, baby, children, so.

I: Why was that? Why do you think was that?

Neil: Enjoying ourselves and you know

Petra: Yeah, I had other things to do with my life.

Neil: having the stress of having a kid and what it brings. And we were married when we were, what? 31. 30, 31. And, you know, busy careers, that’s that’s one thing. But also, because we were enjoying ourself and didn’t want to go into the world of crying and nappies and all that kind of stuff just yet.

I: (laughing)

Neil: So we both said we’d give it a while, which we did. Yeah.

In overlapping speech, Petra and Neil subvert the normative timeline evidenced across our other participant accounts. Petra starts with, ‘we got married but we said’, with Neil overlapping with, ‘we both said’. Marriage seems to be designated as an important milestone, yet the ‘but’ marks out a deviation from what might typically follow. Rather than pursuing the conception of a baby, they co-construct dedicating this time to themselves and to their relationship ‘we wanted to enjoy ourselves’. By distancing herself from the natural order ‘I had other things to do with my life’ and ‘I wasn’t one of the people’ who would follow the expected norm, Petra implies there are alternative identities and life orders to follow.

However, rather than discarding the ‘natural order’, Petra and Neil portray the normative timing of having a baby straight after the marriage, rather than parenthood stage itself, as undesirable. Additionally, by portraying having a baby as a highly demanding and even unpleasant pursuit, they employed the notion of intense parenting (Budds et al., 2016) and presented themselves as responsible parents who only decided to try for a baby when ready to take on the responsibility.

As such the couple demonstrated awareness of the normative timeline, and the pressure to follow it. While their pursuit to ‘enjoy themselves’ was marked as a deviant (unexpected) detour from the normative timeline, they still followed the timeline in the ‘appropriate’ order (marriage, financial/job security, baby). What is portrayed as non-normative is taking time to themselves after the marriage before actively trying for a baby. However, they still implicitly indicate that having a baby was part of their original plan. The prominent normative timeline was thus still present despite this deviation from the expectation, since they explicitly orientated to the normative order.

Our data illustrates the omnipresence of normative parental timelines and how this is socially constructed as a natural step. Therefore, the inability to conceive a child disrupts expectations that the timeline is achievable.

### Balancing the need for social support with problems associated with disclosure

All participants constructed disclosure of trying for a baby, their infertility and fertility treatment as problematic due to the pressure from loved ones to follow the normative timeline. Consequently, conflicts arose regarding social support provided during fertility treatment.

The first extract demonstrates the initial non-disclosure of attempts at natural conception.

#### Extract 3: Diana/Oliver

I: So did anybody else knew or know about your struggles to conceive naturally? Did your family know?

Oliver: Yeah, I think a couple of friends and family. They knew that we were trying long time well and people at work they asking you, ‘Why don’t you have babies together?’

Diana: why do you want marry five years and have no babies and I was like ‘none of your business’

Oliver: So they probably realise that you have got problems. But they weren’t like you know in a bad sense but they were just asking

Diana: Yeah. So that’s the thing that’s the worst part where people were like asking and you know, especially the families when they don’t…we didn’t want to tell them. Like ok we were telling everybody like for the first three years I think that we just waiting for the right moment, waiting for the right moment.

Diana: Until I think my mum broke all that because I had cousins who were struggling to get pregnant as well. They were giving her a really tough time in the family. She was like “It’s been eight years, why do you not have babies? And all that, so she started crying. My mum realised like, okay maybe they can’t. that was about a year and something ago. They just like okay, let it go that’s your time, yeah

Diana: But the worst part was from the family. ‘Is it now? Is it ready?’ Maybe now maybe that’s a good time’ And I was like, oh my god, just leave me alone.

The couples commonly commented on difficulties when dealing with comments regarding their childlessness. Diana/Oliver constructed revealing their fertility problems only to a limited number of friends and family and were regularly subjected to questioning regarding the expectation of having a child. Like many other couples, they resolved the situation by inventing vague reasons regarding the convenient timing for having a child ‘we just waiting for the right moment’ or by rebutting the legitimacy of the question all together ‘none of your business’.

The main pressure came from repeated family questioning about the absence of pregnancy, leading Diana to oppose and disengage ‘oh my god just leave me alone’. This interrogation regarding their childlessness was therefore constructed as bothersome and as contributing to the constant pressure. Diana/Oliver assumed that due to the time delay in following the expected norm, people around them were aware of their difficulties in conceiving. Despite this assumption, our participants rarely disclosed these difficulties to people outside of the few they informed when trying to conceive naturally or at the beginning of the treatment.

Marriage was implicitly portrayed as the starting point in the pursuit of parenthood, which is consistent with existing literature (e.g. Hadley and Hanley, 2011; Webb and Daniluk, 1999). This was affirmed by questioning from people surrounding the couples. Rather than revealing fertility problems, the couples tended to invent vague reasons or to debunk the conversations. These initial rebuttals and pressure to engage in discussions of planned parenthood typically acted as a barrier to later disclosure and the provision of social support during the treatment as we will see later.

The second milestone within the infertility journey involved negotiation of disclosure of the fertility treatment. Although most of the couples, and women specifically, disclosed to a small circle of friends, they still had to deal with wider disclosure.

#### Extract 4: Joana/Steve

Joana: That’s the problem though, once you tell people you’re going for IVF,

Steve: [they wanna know the results

Joana: and [they know ‘oh well she had our eggs put back in’, they wanna know what’s is it what’s the outcome, what’s happening”. We’ve fobbed a few people off, haven’t we (unclear).

We’re still waiting or ignoring people

Joana: But then obviously normally at what would what were we, nearly five weeks pregnant when we found out, normally you wouldn’t tell a soul, you wouldn’t tell anybody, but because people knew that you were trying, you do tell.

Steve: You’ve got to tell the people that know when the egg is going back in or when this is but again there’s not a lot of people

Joana: No we were more open and honest with it (.)

Joana: but for me, the main reason I don’t want to tell people is I can’t I don’t like being pitied. So, the more people you tell, the more people you’ve got to say if it doesn’t work and I don’t want anybody to look at me with that look of pity on their on their face. I can’t cope with that, that’s too too much for me.

Where you’re not stuff like that doesn’t bother you does it

Steve: no because I just tell them to

Joana: it does me stuff like that

Steve: yes but you’re more polite than me

Joana: I think it comes back to disappointing people Does that make sense? I haven’t told my mum yet because I wanted to know what happened at the scan because there’s no point telling her for the scan to be not to be what we want it to be, to have to go and disappoint her (.) Nobody else needs to be upset by the IVF journey when I’ve been upset enough throughout it. Does it make sense?

I: yes

Joana: I’m sure some people are different but for me

Joana: that’s that’s been the hardest thing because obviously you wanna share with people you love and your friends and it’s the time in your life when you probably need more support than than ever, but you also don’t wanna…

Steve: Plus our mums [as soon as you tell ‘em

Joana: [have got big mouths.

Steve: everybody will know so it’s best just not to tell them until we have to.

Our participants explained that providing more detailed insight into the treatment ultimately meant sharing the outcome. In the extract above, disclosure opened a constant dialogue of checking progress, having to disclose treatment failure, and having to deal with the reactions of others regarding the undesirable news. Joana also constructed disclosing bad news as positioning her as somebody to be pitied.

Comparisons to natural pregnancy were typical across our data. An argument was made that it was less common for natural conceptions to be revealed at early stages of pregnancy, whereas if infertility issues were disclosed, then there was an onus to share the news. The comparison to natural pregnancy implies that social disclosure of early pregnancy and pregnancy failure are socially undesirable and non-normative. These conversations may put social and emotional strain on all involved, which is evidenced in this extract by the need to deal with pity. Not only did the couples construct having to work through their own disappointment, but they must additionally deal with the sympathy, upset and disappointment of others. This kind of reaction and social support was therefore seen as highly undesirable, which is consistent with Abraham (2019). One important consequence of non-disclosure was that there was less social support available across treatment stages and failed treatment milestones. We see evidence of this with Joanna’s point ‘it’s the time in your life when you probably *need more support than than ever*, but you also don’t wanna…’ (emphasis added). Whilst what is not wanted is left uncompleted, her assessment is presumably linked to the preceding discussion.

Another barrier to disclosure was the potential that this news was spread to people outside of our participant’s closest social circle as evidenced here by ‘Plus our mums as soon as you tell ‘em.[have got big mouths. everybody will know’. The stigma associated with a delayed normative parental timeline outlined in the first theme is thus extended to a wider social circle and likely to increase the undesirable expressions of pity and sympathy. The disclosure could result in uncomfortable social conversations rather than in social support.

The dilemma in terms of social support is this – it is a delicate balance between the stigma and pressure associated with disclosing versus the acknowledgement of needing support.

#### Extract 5: Suzy/Nathan

Suzy: You have to talk to other people.

Nathan: You have to have that, you can’t do it by yourself.

Suzy: Yeah, and it is tough, and it’s really difficult to have those conversations, but you have to do it for your own, your own sanity in a way.

Both Suzy and Nathan stress that you ‘have to’ talk to other people. However, Suzy (as with most of the participants) highlights the difficulty of those conversations. The impossibility of doing it alone makes the social disclosure an imperative to be able to ‘keep your sanity’. This emphasises how burdensome the treatment is and the benefits of disclosure to the wellbeing of the participants and overall treatment process. Bara, another participant, also noted that disclosing to a few people who understand ‘makes a big, big difference’.

Whilst our participants constructed social support as beneficial, disclosure to a limited number of people who would provide the appropriate social support without the associated problems cited above is one way to resolve the dilemma. Similar to our findings, Maman et al. (2014) found that disclosures of HIV/AIDS status to a trusted family member enabled support, but wider disclosure risked stigma.

### Men’s supportive role

Our data highlighted specific gendered dynamics of support between the partners. As noted earlier, within fertility treatment men take on a supportive role (Hanna and Gough, 2016; Throsby, 2004). However, the dynamics of these roles are unclear.

Fourteen out of 20 of our participants presented the male role as supportive during the treatment in terms of emotional and practical support. Two couples mentioned the man’s financial responsibility for the treatment cost, both women however expressed sadness from the lack of their male partner’s emotional support during the treatment. One couple portrayed the man’s support with injecting as physically harmful. Men were usually constructed as being positive about the possible result whereas women constructed being more cautious.

#### Extract 5: Bina/David

I: Did you discuss this together?

David: Yeah, a lot. Obviously Bina worries a lot and she does bring it to my attention quite a lot. So, most nights we would have a conversation about it. I don’t know why, I was positive again, wasn’t I? I don’t know why. Even before she took the test, I had a I had an inkling or I wasn’t like super positive but I actually thought, well, I’m pretty sure you are. And then she was. And then for me it was a bit of a nightmare because I was like, ‘you just gotta of calm down’. And then you would sort of go into meltdown every now and then and I was like, ‘I can’t understand why you are getting so upset because this is all good not bad’, if you know what I mean so.

Our male participants often talked about having a feeling they knew their partner was pregnant, ‘Even before she took the test, I had a I had an inkling’. Men took on a supportive, comforting role that allowed their partners to offload. Our participants typically did not immediately share treatment progress and results with others, meaning that the emotional support to women dealing with any treatment side effects and worries regarding the outcome was placed upon the male partner. This support was provided continuously and regularly, ‘Obviously Bina worries a lot and she does bring it to my attention quite a lot. So, most nights we would have a conversation about it’. Whilst we cannot generalise based on a small sample, this pattern contrasts with some existing research which argues that men avoid any treatment discussions or decisions (Throsby and Gill, 2004). Studies of online forums where men emphasised the importance of communication, and of being loving to their partner, also often found it difficult to support them emotionally (Hanna and Gough, 2017). Noticeably, our female participants initiated conversations about concerns they had, providing space for male emotional support. This contrast of female concern versus male positivity could create feelings of frustration and misunderstanding in the men as noted by David’s framing of this as ‘a bit of a nightmare because I was like, ‘you just gotta of calm down’. However, overall our female participants constructed male expressions of positivity as emotionally supportive.

#### Extract 6: Bara/Tom

Bara: He’s been good at if he notices that I’ve had a few days of being down, he won’t then let me spend another day in bed sort of thing.

Tom: Yeah.

Bara: Something he’ll be like, ‘Come on, get up, let’s do something’.

Tom: Go to your mam’s or something, or—

Bara: So he’s quite he’s sort of quite good at spotting. And then—

Tom: The thing is, it’s hard because I’ve seen Bara’s sort of mindset going up and down, up and down and like I said, I don’t let it spiral down, I’ll be sort of activated, say, ‘Come on, then, get yoursen dressed, we’re going out somewhere’. Or, ‘Get to your mam’s while I’m at work’. And I think that’s the hard bit, sometimes it’s hard to go off to work and leave her.

Participants presented the man as supportive and in control of their partner’s emotions and wellbeing. Women informed their partner of their emotional wellbeing (see Bina/David). Additionally, the man noticed low emotions in their partner ‘He’s been good at if he notices that I’ve had a few days of being down…’ (Bara/Tom). In both cases, the man was portrayed as proactively managing his partner’s mental and emotional state. Additionally, overseeing her wellbeing implied the man’s responsibility ‘he won’t then let me spend another day in bed sort of thing…….I’ve seen Bara’s sort of mindset going up and down, up and down, and like I said, I don’t let it spiral down…’ (Bara/Tom) and as such created a legitimate role for the man within the treatment.

We theorise that this is due to adherence to traditional masculinity of a proactive man who is in charge and cares for his (distressed) woman. To enable this, the woman needed to present herself in need of support, so somehow struggling. She additionally needed to appreciate her partner’s role as beneficial for her wellbeing and consequently for the whole treatment.

### Conclusion

This study examined how heterosexual couples in the UK managed disclosing their infertility status and sought support seeking during their fertility treatment. In our first theme, the decision to have a child was presented as following a socially normative order and was constructed as the natural next step. This is consistent with other research on infertility (Johansson et al., 2011; Webb and Daniluk, 1999), motherhood (e.g. Budds et al., 2016), and fatherhood (Hadley and Hanley, 2011). Analysis of our deviant case further supported this – despite infertility disrupting this timeline, the normative route was still present within their reproductive story (Jaffe, 2017; Jaffe and Diamond, 2011; Ussher and Perz, 2018). Infertility, and its treatment, were presented as a diversion and incorporated into the anticipated story of parenthood.

A novel contribution is how family and friends socially constructed conception as something natural and easy, placing our participants visibly outside the social norm. Participants demarcated themselves from couples who conceived naturally and this ‘otherness’ could lead to stigma (Goffman, 2009) and potentially increased the couple’s need for non-disclosure.

In our second theme, two stages of infertility disclosure – initial failed attempts at natural conception, and later their infertility and accompanied treatment – were constructed as problematic. The normative route to pregnancy in heterosexual couples (Budds et al., 2016; Johansson et al., 2011; Webb and Daniluk, 1999) and the reproductive story (Jaffe, 2017; Jaffe and Diamond, 2011; Ussher and Perz, 2018) were portrayed by our participants as socially visible, and delays in their timeline lead to questioning, pressure and possible stigma. This resembles research on older motherhood (Budds et al., 2013; Shaw and Giles, 2009), where older mothers were portrayed in the media as abnormal and defective for failing to produce a child within the expected age limits. However, the concept of appropriate stage within a heterosexual relationship to have a baby, in contrast to maternal age, was the signal when to have a baby.

Rather than falling victim to the stigma of infertility (Abraham, 2019) and public dispersal of their inability to conceive (Ussher and Perz, 2018), our participants constructed avoiding widespread disclosure to prevent any future discussions and social pressure. However, this strategy meant that this could act as a barrier to later disclosure of infertility and treatment.

A second stage of disclosure of infertility and treatment was constructed as additionally hindered by having to deal with undesirable emotional responses of others if the treatment cycle failed, and to prevent this news being shared, which is consistent with existing literature (Abraham, 2019; Ussher and Perz, 2018). However, our research supplements extant literature by highlighting how non-disclosure was constructed as problematic even when the couples became pregnant. This was justified by the social conventions regarding early pregnancy, where pregnancy is usually announced post-12 weeks viability scan. This implies that disclosures of early pregnancy and pregnancy failure (natural or post-treatment) are seen as socially undesirable, and consequently poses another barrier in disclosure of fertility treatment and provision of social support.

Our final theme demonstrated how men in our study were constructed as taking on a supportive role, which is consistent with existing research (e.g. Malik and Coulson, 2008; Throsby, 2004; Throsby and Gill, 2004). Most men in our study supported their woman emotionally, and/or during the injections of hormones, which partially reflects the roles outlined by Throsby and Gill (2004). Whilst we cannot generalise from a small sample, our findings contradict previous research (e.g. Hanna and Gough, 2016; Throsby, 2004; Throsby and Gill, 2004) by proposing that men found emotional support difficult due to the traditional masculinity of un-emotional man. Our data suggested a pattern in the provision of support:

- The woman presented herself as struggling and in need of support.
- Their partner stepped in and took care of their woman, comforting and supporting her.
- The man continuously monitored ‘his woman’, taking control of her wellbeing.
- The woman appreciated and praised their partner’s support.

We suggest that this pattern draws on a traditional chivalrous masculinity (Girouard, 1981) of a loyal, brave (and proactive) man who protects his woman. However, for this pattern to be effective, the woman needed to portray herself as struggling and in need of support. This emphasises the initial role of a woman in creating a legitimate space for the man during the treatment to support them. It can also help explain research findings on male online infertility forums, where men claimed difficulty dealing with their partner’s emotions when she pushed them away (Malik and Coulson, 2008) and consequently did not create space or need for male support. This could be something that clinicians raise with their patients. This theme also adds to limited literature on marital benefits of undergoing infertility treatment in relation to the development of good communication and supportive behaviour that both partners saw as beneficial (Sauvé et al., 2020).

We also found additional dichotomy of roles that were constructed – women were positioned as cautious and worried during treatment process, while the men were positive about specific treatment stages. This is consistent with studies on male online support groups (Hanna and Gough, 2017; Malik and Coulson, 2008). However, our study demonstrated this from the perspective of both partners (rather than men only), where the men acted as a comforting force for the women.

### Implications, limitations and future research

Our study has implications for infertility professionals and counsellors, for patients and the wider population. We found that barriers to disclosure of treatment and accompanied social support start early on with first failed attempts for natural conception. This initial non-disclosure prevents the couples from accessing social support later during the treatment when they most need it. We argue that infertility clinicians could alert patients early on to the likelihood of prolonged exposure to social pressure surrounding conception. Strategies on how to deal with the pressure and on how to safely disclose and negotiate social support would be beneficial. Change of societal norms around discussions of failed attempts in conception and early pregnancy via more education would be valuable.

Another unique finding is the important role of the woman in openly expressing their struggles and need for help, thus affording space for a male protective role within the treatment. Awareness of these dynamics could encourage couples to allocate valid places for the male supportive role during the treatment within the framework of traditional masculinity and femininity and ultimately improve the wellbeing of couples during the treatment. However, we are aware that our findings and interpretations are based on a small sample size inviting further investigations into the dynamics of support within heterosexual couples undergoing infertility treatment.

Our study investigated a seldomly heard participant population. We interviewed patients in a very sensitive stage of their IVF or IVF/ICSI treatments and lives where all but two of them were interviewed shortly after their treatment cycle and pregnancy test. We are aware that inclusion of the historic interviews could allow for different couples’ dynamics during interviews. However, this still allowed us to investigate negotiations of social support, treatment disclosure and the dynamics of gender roles during the treatment. Participants were recruited at the start of their IVF or IVF/ICSI treatment when we could not predict their treatment results. At the time of interview, most couples were in very early stages of pregnancy, but three of our couples were not pregnant. Due to difficulties in reaching the target population, the type of infertility (male, female, mixed, unexplained) was not accounted for in the current study. Thus, future research could investigate participant groups according to different types and causes of infertility thus adding clarity on if and how the cause of infertility influences identified gender roles and communication during treatment. Additionally, division according to treatment results could also yield further findings. Our study confirmed the male supportive role within the treatment. However, future research could investigate how men deal with their own worries and their own support. Our study provided more insight into the dynamics of disclosure and social support during fertility treatment and into roles within heterosexual dyads during fertility treatment.
